# The prevention of injuries among youth basketballers according to the “Sequence of Prevention”: a systematic review

**DOI:** 10.17159/2078-516X/2021/v33i1a10829

**Published:** 2021-01-15

**Authors:** D Aarts, M Barendrecht, E Kemler, V Gouttebarge

**Affiliations:** 1 Avans+ Improving Professionals, Breda, the Netherlands; 2 Dutch Consumer Safety Institute, Amsterdam, the Netherlands; 3 Amsterdam UMC, University of Amsterdam, Department of Orthopaedic Surgery, Amsterdam Movement Sciences, Meibergdreef 9, Amsterdam, the Netherlands; 4 Section of Sports Medicine, University of Pretoria, Pretoria, South Africa; 5 Amsterdam Collaboration on Health & Safety in Sports (ACHSS), Amsterdam UMC IOC Research Center of Excellence, Amsterdam, the Netherlands

**Keywords:** basketball, injury, risk factor, preventive intervention

## Abstract

**Background:**

Basketball is played by the youth worldwide, and various injuries occur in youth basketball. There is currently no overview of the incidence, the risk factors and preventive measures of musculoskeletal injuries among youth basketball players.

**Objective:**

This systematic review describes the most common injuries among youth basketball players. The most common risk factors and various preventive measures and interventions have also been reported and discussed.

**Methods:**

Search strategies were built based on groups of keywords, namely ‘injury’, ‘youth basketball’, and ‘cohort’. Search strategies were entered into Medline and SPORTDiscus. Titles, abstracts and full text articles were screened by two researchers. Data from the included articles were extracted by one researcher and checked by another researcher.

**Results:**

Twenty-seven studies showed that the overall injury rate ranged from 2.64 to 3.83 per 1 000 hours of exposure. Ankle-(22%–37%) and knee injuries (5%–41%) were the most common injuries. Risk factors for knee injuries included ankle dorsiflexion with a range less than 36.5 degrees and female athletes with greater hip abduction strength. High variations of postural sway corresponded to occurrences of ankle injuries (p=0.01, OR =1.22; p<0.001, OR =1.22). A core intervention (rate = 4.99/1 000 athlete exposure (AEs)) focused on the trunk and lower extremity led to a reduction in injuries compared to a sham intervention (rate =7.72/1 000 AEs) (p=0.02). Wearing a McDavid Ultralight 195 brace reduced ankle injuries compared to the controls (HR 0.30; 95 % CI 0.17 0.90; p=0.03).

**Conclusion:**

Ankle and knee injuries are the most common injuries among youth basketball players. Poor postural control, reduced ankle dorsiflexion and high hip abduction strength are the main risk factors. A neuromuscular warm-up, in combination with strength and stability exercises, seems to be the best training method to prevent injuries. Ankle injuries can be reduced by wearing a lace-up ankle brace.

Youth play basketball all over the world. There are approximately 450 million youth basketball players worldwide. ^[1]^ In the USA, basketball was the most popular team sport for boys (544 811) and girls (457 986) registered in the school year of 2003–2004.^[2]^ Nearly 975 000 American students participated in secondary school basketball during the 2015–2016 academic year.^[3]^ In 2000–2001, basketball was the most common cause of sports- and recreation-related injuries seen in USA emergency departments, with a total of 395 251 cases.^[4]^ The proportion of cases was not evenly distributed across age groups – for boys aged 5–9 years, basketball accounted for 5% of all sports injuries, whereas it constituted 15% of cases for boys aged 10–14 years, and 26% for boys aged 15–19 years, the highest percentage for any activity in this group.^[4]^ For girls aged 10–14 years, basketball was responsible for 15% of all sports injuries, and 18% in the 15–19 years age group.^[4]^In a cross-sectional study in Canada among 1466 students (12–15 years old), the greatest proportion of sports injuries occurred in basketball (14%).^[5]^ There have also been basketball injuries in Africa. For example, in Rwanda, one study showed that the injury rate was 3.6 injuries per player per season, while in another study in Ghana, the injury incidence was 0.190 and 0.084 per 100 players during competition and training.^[6,7]^ In a study which focused on several sports, basketball athletes were reported to have the highest injury rate.^[8]^ Overall the incidence rates for basketball are higher during matches than in training sessions.^[9]^ Results for adolescent basketball players revealed that the injury incidence varied considerably from 7.8 – 49.0 per 100 participants for girls and 5.6 – 36.8 per 100 participants for boys.^[9]^

The National Collegiate Athletic Association (NCAA) began collecting injury and exposure data in 1982.^[10]^ By summarising the data from all sports, the injury cases were significantly higher in matches than in training sessions, and pre-season training session injury rates were significantly higher than both in-season and post-season training session rates.^[10]^ There were no changes in the rates of injury over the 16 years they collected injury data. More than 50% of all injuries were to the lower extremity, with ankle ligament injuries being the most common among male basketball players.^[10]^ Marchi et al. reported in 1999 that 23% of the ankle sprains among children aged 6 to 15 years resulted in permanent complaints over 12 years of follow-up.^[9]^ In the USA, emergency department visits are highest among school-age children.^[12]^ Over one-third of school-age children will sustain an injury sufficiently severe to be treated by a doctor or nurse.^[12]^ Besides personal suffering, high healthcare costs are also incurred. Youth basketball is also responsible for many injuries in Europe: after football, basketball is the sport responsible for the highest number of injuries among boys.^[13]^

The extent of the injury problem in youth basketballers calls for preventive action based on the results of epidemiological research. The number of injuries among youth basketball players should be reduced to prevent long-term complaints or complaints into adulthood and to reduce healthcare costs. The ‘van Mechelen sequence of prevention model for sports injuries’ describes four consecutive steps that lead to efficacious preventive interventions. ^[14,15]^ Steps 1 and 2 consist of exploring the incidence and aetiology of musculoskeletal injuries. Steps 3 and 4 consist of developing and evaluating preventive interventions.^[14]^ A systematic review on van Mechelen’s quadrants in adult basketball players has already been published, but not yet in youth basketball players.^[16]^ Therefore, we aimed to gather epidemiological information to answer the following questions: (1) What is the incidence of musculoskeletal injuries among youth basketball players?; (2) What are the risk factors of these musculoskeletal injuries among youth basketball players?; (3) What are the interventions available for the prevention of musculoskeletal injuries among youth basketball players?; and (4) How effective are these interventions on the reduction of musculoskeletal injuries among youth basketball players?

## Methods

### Design

A systematic review was conducted on sports injury prevention among youth basketball players. This systematic review has been written in accordance with the PRISMA guidelines.^[17]^

### Data sources and searches

Search strategies (Appendix 1) were composed by using three groups of keywords, namely: ‘injury’, ‘youth basketball’, and ‘cohort study’. The search strategies were entered into two databases, namely Medline and SPORTDiscus. Medline was searched from October 2, 2018 up to February 7, 2019. SPORTDiscus was searched from October 2, 2018 up to January 8, 2019. Different filters were used: Humans, English, Randomised Controlled Trial, Systematic Review and/or Academic Journal. All search terms were combined with ‘AND’ and ‘OR’.

### Eligibility criteria

The inclusion criteria were:

- The population consists of youth (boys and/or girls) basketball players (age 6–18).
- The article is written in English.
- If the article is about descriptive epidemiology (Step 1 of van Mechelen’s model), prospective cohort design is used.
- If the article is about descriptive epidemiology, incidence rates or prevalence rates are reported.
- If the article is about aetiology (Step 2 of van Mechelen’s model), prospective cohort design is used.
- If the article is about aetiology, a risk estimate is reported.
- If the article is about prevention (Steps 3 and 4 of van Mechelen’s model), randomised controlled trial is conducted.
- If the article is about prevention, incidence rates and/or effect sizes are reported.

### Study selection

Titles and abstracts of the retrieved citations were independently screened by two researchers (DA and VG). When the title and abstract met the inclusion criteria, the article was included for the full text selection. When the title and abstract did not contain sufficient information, it was not included for the full text selection. Then the full text articles were independently assessed by two researchers (DA and JA). Where doubts arose concerning inclusion or exclusion of an article, a third researcher was consulted (MB).

### Data extraction

The data from the included articles were extracted by one researcher (DA) in a standardised table and checked by another researcher (MB). The data extraction focused on: article information (author, year), study population (numbers, age and gender), injury definition and injury incidence. If there was information about the risk factors, preventive measures and the effect of these preventive measures, it was also included in the data collection.

### Risk of bias appraisal

To assess the methodological quality of the included articles, two different checklists were used. For the articles related to descriptive epidemiology and aetiology, the Quality in Prognosis Studies (QUIPS) tool was used (Appendix 2). The Cochrane Collaboration’s tool was used for the articles related to prevention (Appendix 2). For both the QUIPS and the Cochrane Collaboration’s tool, six potential bias domains were assessed with a high, moderate or low risk of bias. For assessments using the QUIPS tool, a study was considered to have a low risk of bias rated as low or moderate in all six domains, with at least four domains being rated as low.^[17]^ If two or more domains were scored as high, the study was rated as having a high risk of bias.^[17]^ Studies that were in between were scored as having a moderate risk of bias.^[18]^ For assessments using the Cochrane Collaboration tool, a study was assessed with a low risk of bias when all items were assessed as low.^[18]^ When at least one item was assessed as moderate, the article received a score with a moderate risk of bias. A high risk of bias was rated when at least one item was assessed as high.^[18]^ The checklists were assessed and cross-checked by two researchers (DA and JA). If a difference of opinion arose concerning the scoring of an item, a consensus was reached.

### Data synthesis and analysis

The data were processed according to the four steps of van Mechelen’s ‘Sequence of Prevention’ so that the collected information was presented clearly. The following outcome measures were used for the incidence and the risk factors: exposure, hours of exposure, hours of game exposure, athlete exposure, and percentages. Only the results that were expressed in hours of exposure were included in the results of the overall injuries. For the effectiveness of the preventive measures, it was considered whether a reduction was found on these outcome measures. Risk reduction rates were used.

## Results

### Search strategies

**Fig. 1:**
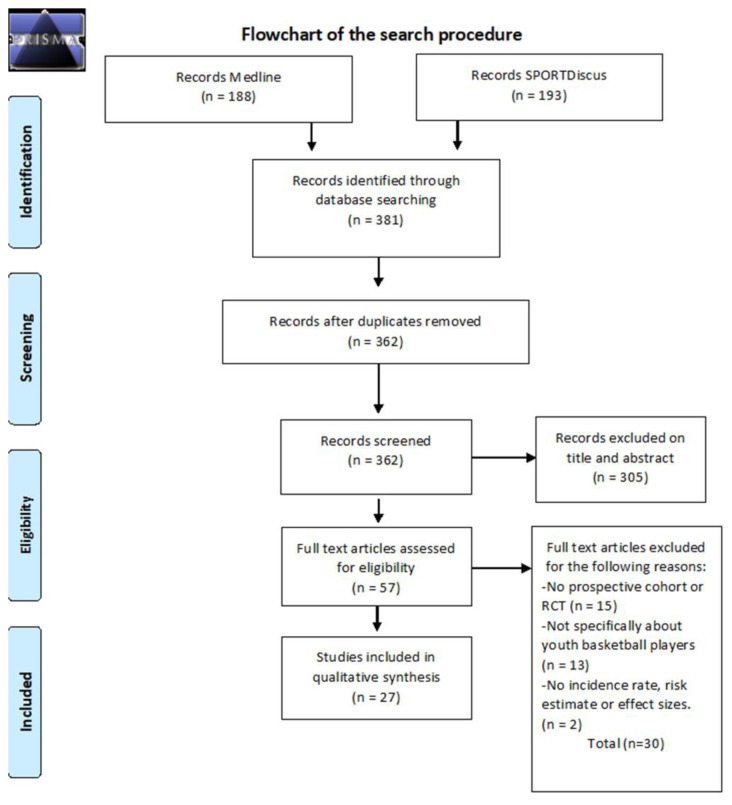
Flow chart of the search procedure

A total of 381 citations were identified of which 188 were in Medline and 193 in SPORTDiscus. Of the 381 relevant citations, 19 were duplicates. After checking the titles and abstracts against the inclusion criteria, 57 potentially relevant studies were included for the full text review.^[20–76]^ After screening the full text, 30 articles were excluded.^[20–49]^ The reasons for exclusion were: the articles did not meet the requirements for a prospective cohort or an RCT (n = 15) ^[20–22,28,30,31,34–36,39–43,48]^, the articles were not specifically about youth basketball players (n = 13) ^[21,23–26,29,32,37,38,44–47]^, or there were no outcome measures (n = 2).^[27,33]^ Figure 1 presents the search procedure.

### Risk of bias

Of the 27 included studies, 23 studies were checked using the QUIPS tool (descriptive epidemiology and aetiology) ^[49–71]^ and four studies were checked using the Cochrane Collaboration’s tool (prevention).^[73–76]^ Fifteen of the 27 studies scored a low risk of bias.^[49,50,56,58,59,63–65,67,69–72,74,75]^ The other 12 studies scored a moderate risk of bias.^[51–55,57,60–62,66,68,73]^ An overview of the scores can be found in Table 1.

### The incidence of musculoskeletal injuries in youth basketball

Of the 27 included articles for data extraction, 19 articles contained information on the incidence of musculoskeletal injuries in youth basketball. ^[49–67]^ Of these 19 studies, 13 studies were about girls and boys, five studies were about girls only and one study was about boys only. The age of all participants in these 19 studies together ranged from 8 to 20 years. Twelve studies were about American basketball players and seven studies were conducted in other countries (five in Europe, one in Japan and one in Nigeria).

#### Overall injuries

The overall injury rate for youth basketball players ranged from 2.64 to 3.83 per 1 000 hours of exposure.^[50,51,56]^ The game injury rate (range 5.70 to 36.84 per 1 000 hours of game exposure) was higher than the practice injury rate (range 1.47 to 3.13 per 1 000 hours of exposure).^[50,51,55,56]^ Ankle injuries (range 22% to 37%) and knee injuries (range 5% to 41%) were the most common injuries.^[50,53,54,56,57]^ Sprains were the most common type of musculoskeletal injuries overall (ranging from 43% to 66%), followed by fractures (ranging from 4% to 12%). ^[52–54,57]^ Information on the incidence of injuries is presented in Table 2, Appendix 3 and Appendix 5.

#### Specific injuries

McGuine et al. investigated the incidence of ankle sprains in youth basketballers; the rate of ankle sprain was 1.56 per 1 000 exposure (1.68 for boys and 1.44 for girls per 1 000 exposure).^[63]^ Two studies investigated the incidence of patellofemoral pain (PFP) in female youth basketball players. Herbst et al. found an incidence rate for development of PFP of 0.97 per 1 000 athlete exposures (AE), the study of Myer et al. found an incidence rate of 1.09 per 1 000 AE. ^[65,66]^ Symptoms of anterior knee pain were likely to persist to after middle school-aged onset and to reach peak prevalence during the high school years.^[68]^ The shoulder injury rate ranged from 0.045 to 0.061 per 1 000 AE. ^[60,61]^

### Risk factors of musculoskeletal injuries in youth basketball

Eleven articles presented information on the risk factors of musculoskeletal injuries in youth basketball. ^[50,51,58,59,63,65,66,68–71]^ Of these 11 studies, five were about girls and boys, four were about girls only and two studies were about boys only. The age of the participants in these 11 studies together ranged from 9 to 20 years. In seven of the 11 studies, research was done on American youth basketballers. In the other four studies, research was done in different countries (three in Europe and one in Taiwan).

#### Game vs practice

The injury risk for basketball injuries in youth was higher in games than in practices in all included studies. Most game injuries resulted from body contact, 46% in the study of Kuzuhara et al. and 1.32/1 000 AE for boys and 1.55/1 000 AE for girls in the studies of Clifton et al. ^[51,58,59]^ In the study of Pasanen et al. body contact with another player was the most frequent injury situation (25%), followed by stepping or landing on another player’s foot (23%) or landing from a jump (16%). Proportions of contact injuries, indirect contact injuries, and non-contact injuries were 49%, 17%, and 34%, respectively.^[50]^ Information on the aetiology of injuries is presented in Table 2, Appendix 3 and Appendix 5.

#### Risk factors of lower extremity injuries

An anterior right/left reach distance difference measured with the Star Excursion Balance Test (SEBT) is a risk factor for lower extremity injuries in youth basketball players.^[69]^ Logistic regression models indicated that players with an anterior right/left reach distance difference greater than four cm were two and a half times more likely to sustain a lower extremity injury (p<0.05).^[69]^ Girls with a composite reach distance less than 94% of their limb length were six and a half times more likely to have a lower extremity injury (p<0.05).^[69]^

#### Risk factors of ankle injuries

In boys’ high school basketball players, high variations of postural sway in one leg standing is a risk factor for developing an ankle injury.^[70]^ Postural sway was assessed through standing performance on one leg with open eyes and was measured on a force plate. High variations of postural sway in both anteroposterior and mediolateral directions corresponded to occurrences of ankle injuries (p=0.01, odds ratio [OR] =1.22; 95 % confidence interval [CI] 1.046–1.424; p<0.001, OR =1.22; 95 % CI 1.089–1.359).^[70]^ Subjects who demonstrated poor balance (high sway scores) had nearly seven times as many ankle sprains as subjects who had good balance (low sway scores) (p<0.001).^[63]^

#### Risk factors of knee injuries

Players with an ankle dorsiflexion range less than 36.5 degrees had a risk of 19% to 29% of developing patellar tendinopathy (PT) within a year, compared with 1.8% to 2.1% for players with an ankle dorsiflexion range greater than 36.5 degrees.^[71]^ The ankle dorsiflexion was measured with the established weight-bearing lunge test. Young female basketball athletes with greater hip abduction strength have an increased risk for the development of PFP-need to write this out in full first. Female athletes who developed PFP demonstrated increased normalised hip abduction strength (normalised torque, 0.013 ± 0.003) relative to the referent control group (normalised torque, 0.011 ± 0.003) (*P* <0.05).^[65]^ Additionally, frontal plane loads contribute to increased incidence of PFP in young female basketball athletes.^[66]^

### Preventive interventions and related effectiveness

Four studies regarding injury-preventive interventions were included. ^[72–75]^ Foss et al. investigated the effects of a school-based neuromuscular training (NMT) programme on sports-related injury incidence at high school and middle school levels, focusing particularly on knee and ankle injuries.^[73]^ The NMT intervention (CORE group) consisted of exercises focused on the trunk and lower extremity. The NMT intervention consisted of the following thirteen exercises: lateral jump and hold; step hold; BOSU swimmers; BOSU double-knee hold; single-legged lateral AIREX hop-hold; single tuck jump with soft landing; front lunges; lunge jumps; BOSU (flat) double-legged pelvic bridges; single legged 90 degrees hop hold; BOSU lateral crunch; box double crunch; Swiss ball back hyperextensions. The control intervention (SHAM group) consisted of resisted running exercises using elastic bands. For basketball, the athletes in the CORE group (rate = 4.99 injuries/1 000 AEs) demonstrated lower injury incidences than the athletes in the SHAM group (rate =7.72 injuries/1 000 AEs) p = 0.002.^[72]^ The CORE group showed a reduction in injuries for basketball players (p = 0.02).^[72]^ The absolute risk reduction rate per 1 000 AEs was: 2.73 (95% CI 0.92, 4.54).^[72]^

LaBella et al. evaluated the effectiveness of coach-led neuromuscular warm-up on reducing lower extremity injuries in young female soccer and basketball athletes.^[73]^ The warm-up was similar to previously studied NMT programmes, combining progressive strengthening, plyometric, balance, and agility exercises. Athletes were instructed to avoid dynamic knee valgus and to land from jumps with flexed hips and knees. Coaches for the control group used their usual warm-up. Compared to controls, athletes in the intervention group had lower incidence rates per 1 000 AEs of gradual-onset lower extremity injuries (0.43 vs 1.22, p<0.01), acute-onset non-contact lower extremity injuries (0.71 vs 1.61, p<0.01), non-contact ankle sprains (0.25 vs 0.74, p=0.01) and lower extremity injuries treated surgically (0.00 vs 0.17, p=0.04).^[73]^

Emery et al. studied the effectiveness of a sports-specific balance training programme in reducing injury in adolescent basketball.^[74]^ The training group and the control group were taught a standardised warm-up programme. The training group was also taught an additional warm-up component and a home-based balance training programme using a wobble board. The injury rate in the control group was 33.1 injuries per 100 participants per season (95% CI; 28.64–37.79); in the training group it was 26.3 injuries per 100 participants per season (95% CI; 22.48–30.43).^[74]^ The basketball-specific balance training programme was protective with regard to acute onset injuries in high school basketball (RR = 0.71 95% CI; 0.5–0.99),^[71]^ but not significant, like all the results from this study. Self-reported compliance to the intended home-based training programme was poor (60%).^[74]^

McGuine et al. investigated the effect of lace-up ankle braces on the incidence and severity of acute first-time and recurrent ankle injuries sustained by high school basketball players.^[75]^ Athletes were instructed to wear McDavid Ultralight 195 braces over a single pair of socks on both ankles for each team-organised conditioning session, practice, or competition throughout the season. The rate of acute ankle injuries was 0.47/1 000 exposures in the braced group and 1.41/1 000 exposures in the control group (Cox hazard ratio [HR] 0.32; 95% CI 0.20–0.52; p=<0.001).^[75]^ For players with a previous ankle injury, the incidence of acute ankle injury was 0.82/1 000 exposures in the braced group and 1.79/1 000 exposures in the control group (Cox HR 0.30; 95% CI 0.17–0.90; p=0.028).^[75]^ Information about the preventive interventions and effectiveness is presented in Table 3, Appendix 4 and Appendix 5.

## Discussion

The results showed that the overall injury rate for youth basketball players ranged from 2.64 to 3.83 per 1 000 hours of exposure.^[50,53,56]^ Ankle injuries (22%–37%) and knee injuries (5%–41%) were the most common injuries.^[50,51,54,56,57]^ Several risk factors for developing these injuries were mentioned in the Results section, including an anterior right/left reach distance difference, high variations of postural sway and an ankle dorsiflexion range of less than 36.5 degrees.

The anterior right/left reach distance and the variations of postural sway were both measured with a static balance on one leg. This may indicate that poor static balance on one leg, in particular, is a predictor of developing lower extremity injuries in youth basketball players.

For the preventive measures, results showed that a CORE intervention focused on the trunk and lower extremity led to a reduction in injuries (p=0.02).^[72]^ The basketball-specific balance training programme was protective with regard to acute-onset injuries in high school basketball.^[74]^ Another effective preventive measure was wearing a McDavid Ultralight 195 brace. This brace reduced acute ankle injuries.

### Similarities and differences with other studies

In 16 of the 27 included articles, research was done on American youth basketball players. The rules of the basketball federation in America (NBA) and the rules of the basketball federation in Europe (FIBA) are different. The study of Madarame suggested that women’s basketball games are played in a different manner in each region of the world.^[76]^ This could mean that the incidence and risk factors of injuries are different in each region of the world for women/girls. For European basketball girls, and probably also boys, more research must be done into incidence, aetiology and preventive measures.

Compared with the overall injury rates in adult basketball players (ranged from 0.05 to 12.92 per 1 000 hours of exposure),^[16]^ the overall injury rate in youth basketball players (2.64 to 3.83 per 1 000 hours of exposure) is less and closer together. However, in the review of Kilic et al. there is only one article that describes the incidence in 1 000 hours of exposure. In accordance with the review of Kilic et al. ^[16]^, the ankle and knee are the most common injuries in youth basketball. For ankle injuries, a high postural sway was a risk factor in both reviews.^[16,70]^ Because ankle and knee injuries are most common among the youth and adults, it seems best to reduce these injuries in the youth. It would therefore be best to start with preventive measures for the youth and to adjust the corresponding youth exercises for adults later.

The injury rate in games was higher than in practice in all included studies. This is similar to other sports.^[9]^ In youth basketball, body contact is the main reason for injuries in games.^[50]^ At training sessions, there are forms of practice with no body contact. The competitive pressure will probably also be a reason for more injuries in games and this should be investigated in future studies.

### Methodological aspects

It was hard to compare the findings between studies because the denominator varied from 1 000 person-days to 1 000 exposures and athletes per season. For the results of the overall injury rates, only results expressed in hours of exposure were included. The influence that this can have on the overall injury rates seems to be small because the studies with other outcome measures show roughly the same results. In future, it is advisable to use one outcome measure for all studies reporting epidemiological data. Increasingly, incidence rates in all sports are being expressed as rates per 1 000 hours. This is a good approach and allows some comparison across sports.^[77]^ It is better than per exposure because not every individual takes part in a training session or game for an equal period of time.

Some limitations need to be addressed. The search for articles was done in two databases and only articles written in English were included. In this review, only studies with a prospective design were included to formulate valid answers to the research questions while maintaining the highest scientific quality. The databases and selection criteria used might have led to the exclusion of relevant studies with a different design or in another language. We believe that these limitations did not significantly affect the findings, because the two databases used were the most obvious ones and prospective studies were used to formulate valid answers.

There is a lack of scientific literature on the aetiology of basketball-specific shoulder and lower back injuries among youth basketball players. The search for articles only revealed articles about the aetiology and prevention of ankle and knee injuries. Studies on the aetiology and prevention of other specific regions of the body are lacking. Little is known about the incidence of injuries to other regions of the body and this information is presented in Table 2. Because there are no studies into the aetiology of those injuries, prevention studies cannot be drawn up for these few common injuries.

Several risk factors for injuries in youth basketball players were found in this overview. Conclusions on the risk factors for youth basketball injuries are derived from only one study. Therefore, it is important to be cautious when interpreting these risk factors. More research into risk factors for youth basketball injuries is recommended.

In some studies, the ages of the children used were not specifically described and in other studies an average age was used. In two studies, the maximum age of the included participants was 20 years. ^[49,71]^ Because participants older than 18 years were a small group of the participants of all included articles, we believe that our results are still representative of youth basketball players.

All included studies scored a low or moderate risk of bias. This means that we must be careful with some of the conclusions. This systematic review scores a level of evidence 2. A level of evidence 2 applies to the results of the incidence and risk factors. A level of evidence 1 applies to the conclusions from the studies on preventive measures, except for the study by LaBella et al., which scores a level of evidence 2.

### Implications for practice

Based on the results from the included studies on risk factors, it seems advisable to do a screening at the start of the season for ankle mobility, the strength of the hip abductors and the reach distance of the lower extremities. If there are risk factors, they will have to be addressed in a preventive program to prevent injuries.^[78]^ Also, it is advisable to perform a neuromuscular warm-up in combination with performing weekly strength and stability exercises for the trunk and lower extremity and to wear a lace-up ankle brace around both ankles in each training session and each game.

## Conclusion

The conclusions of this systematic review are predominantly based on American youth basketball players, showing an overall injury rate ranging from 2.64 to 3.83 per 1 000 hours of exposure. Ankle and knee injuries were the most common injures among youth basketball players. The main risk factors for injuries in youth basketball were: playing games, anterior right/left reach distance difference greater than four cm, an ankle dorsiflexion range less than 36.5 degrees and high variations of postural sway in one leg. Physical therapists and coaches can use the SEBT to identify youth basketball players who are at increased risk for a lower extremity injury. The hip strength and the ankle dorsiflexion can also be tested in knee complaints or to prevent PT and PFP. A neuromuscular warm-up in combination with performing weekly strength and stability exercises for the trunk and lower extremity currently seems to be the best training method for preventing injuries in youth basketball players. Acute ankle injuries can be reduced by wearing a McDavid Ultralight 195 brace.

For the preparation of specific prevention programmes in youth basketball, further research on the incidence and especially on the aetiology is needed.

## Supplementary Information



## Figures and Tables

**Table 1:** Risk of bias appraisal

Study (Questions 1 & 2)	Participation	Attribution	Prognostic	Outcome	Confounding	Analysis	Total risk of bias
**Backman 2011** ^[71]^	Low	Moderate	Low	Low	Low	Low	Low
**Backx 1991** ^[55]^	Moderate	Moderate	N/A	Low	N/A	Low	Moderate
**Beynnon 2005** ^[62]^	Moderate	Moderate	N/A	Low	N/A	Low	Moderate
**Bonza 2009** ^[61]^	Moderate	Moderate	N/A	Low	N/A	Low	Moderate
**Clifton 2018** ^[58]^	Low	Moderate	N/A	Low	N/A	Low	Low
**Clifton 2018** ^[59]^	Low	Moderate	N/A	Low	N/A	Low	Low
**Field 2011** ^[68]^	Moderate	High	N/A	Low	N/A	Low	Moderate
**Foss 2012** ^[67]^	Low	Moderate	N/A	Low	N/A	Low	Low
**Gomez 1996** ^[54]^	Low	High	N/A	Low	N/A	Low	Moderate
**Herbst 2015** ^[65]^	Low	Low	Low	Low	Moderate	Low	Low
**Kuzuhara 2016** ^[51]^	Low	High	Low	Low	Moderate	Low	Moderate
**Leppänen 2017** ^[49]^	Low	Moderate	N/A	Low	N/A	Low	Low
**McGuine 2000** ^[63]^	Low	Moderate	Low	Low	Moderate	Low	Low
**Messina 1999** ^[53]^	Moderate	Moderate	N/A	Low	N/A	Low	Moderate
**Myer 2010** ^[66]^	Low	Moderate	Moderate	Low	Moderate	Low	Moderate
**Owoeye 2012** ^[57]^	Moderate	Moderate	N/A	Moderate	N/A	Low	Moderate
**Pasanen 2017** ^[50]^	Low	Moderate	N/A	Low	N/A	Low	Low
**Pilsky 2006** ^[69]^	Low	Moderate	N/A	Low	N/A	Low	Low
**Rechel 2008** ^[52]^	Moderate	Moderate	N/A	Low	N/A	Low	Moderate
**Robinson 2014** ^[60]^	Moderate	High	N/A	Low	N/A	Low	Moderate
**Rossi 2018** ^[64]^	Low	Moderate	N/A	Low	N/A	Low	Low
**Wang 2006** ^[71]^	Low	Low	Low	Low	Moderate	Low	Low
**Yde 1990** ^[56]^	Low	Moderate	N/A	Low	N/A	Low	Low

**Study (Questions 3 & 4)**	**Sequence**	**Allocation**	**Blinding**	**Incomplete**	**Selective**	**Other**	**Total risk of bias**

**Emery 2007** ^[74]^	Low	Low	N/A	Low	Low	Low	Low
**Foss 2018** ^[72]^	Low	Low	N/A	Low	Low	Low	Low
**LaBella 2011** ^[73]^	Low	Moderate	Moderate	Low	Low	Low	Moderate
**McGuine 2011** ^[75]^	Low	Low	N/A	Low	Low	Low	Low

Questions: (1) What is the incidence of musculoskeletal injuries among youth basketball players?; (2) What are the risk factors of these musculoskeletal injuries among youth basketball players?; (3) What are the interventions available for the prevention of musculoskeletal injuries among youth basketball players?; and (4) How effective are these interventions on the reduction of musculoskeletal injuries among youth basketball players?

**Table 2:** Musculoskeletal injuries among youth basketball players: occurrence and aetiology

Reference	Total	Boys total	Girls total	Game	Practice	Lower extremity	Ankle	Knee
Leppänen ^[49]^ IR in 1 000 h of exp.	1.51 (95%CI 1.20 – 1.82)	1.20 (95%CI 0.86 – 1.62)	1.93 (95%CI 1.43 – 2.56)				0.07 (95%CI 0.02 to 0.16)	0.59 (95%CI 0.42 to 0.81)
Pasanen ^[50]^ IR in 1 000 h of exp.	2.64 (95%CI 2.23 – 3.05)			Girls: 32.43 (95%CI 22.01 – 42.85) Boys: 36.84 (95%CI 24.86 – 48.82)	Girls: 1.56 (95%CI 1.06 – 2.05) Boys: 1.47 (95%CI 1.06 – 1.88)		15.05 (95%CI 9.79 – 20.31)	6.80 (95%CI 3.25 – 10.34)
Kuzuhara ^[51]^ IR in 1 000 AHs	3.83 (95%CI 3.04 – 3.87)			12.92 (95%CI 7.52 – 18.32)	3.13 (95%CI 2.39 – 4.62)	0.93 (95%CI 0.54 – 1.32)		
Rechel ^[52]^ IR in 1 000 AE				Girls: 3.60 RR 2.63 (95%CI 2.15 – 3.22) Boys: 2.98 RR 2.05 (95%CI 1.69 – 2.49)	Girls: 1.37 Boys: 1.46			
Messina ^[53]^ IR in athlete per season		0.56	0.49	Injury risk girls: 16.0 Injury risk boys: 16.9	Injury risk girls: 2.0 Injury risk boys: 1.8			Girls: 0.1 per athlete year. Boys: 0.06 per athlete year. Injury risk girls: 0.71 Injury risk boys: 0.31
Gomez ^[54]^ IR in athlete per season	0.49						N= 135 (31%)	N= 86 (19%)
Backx ^[55]^ IR in 1 000 hours				23.0				
Yde ^[56]^ IR in 1 000 playing hours	3.0			5.7	2.4		33%	5%
Owoeye ^[57]^ IR per 100 participants or per match	22.7 per 100 participants			Overall: 1.0 per match. Girls: 0.9 per match. Boys: 1.1 per match.			Total: N= 7 (21.9%) Girls: N= 3 Boys: N= 4	Total: N= 13 (40.6%) Girls: N= 6 Boys: N= 7
Clifton ^[58]^ IR in 1 000 AE		1.55					Competition: 0.85 Practice: 0.39	Competition: 0.33 Practice: 0.12
Clifton ^[59]^ IR in 1 000 AE			1.82				Competition: 0.98 Practice: 0.33	Competition: 0.66 Practice: 0.19
Robinson ^[60]^ Shoulder IR in 10 000 AE		0.50	0.61	Girls: 1.24 Boys: 0.95	Girls: 0.34 Boys: 0.32			
Bonza ^[61]^ Shoulder IR in 10 000 AE		0.47	0.45	Girls: 0.76 Boys: 0.90	Girls: 0.32 Boys: 0.30			
Beynnon ^[62]^ Ankle IR in 1 000 person-days		0.42	1.90				Girls: 1.90 Boys: 0.42	
McGuine ^[63]^ Ankle sprain IR in 1 000 exp.	1.56	1.68	1.44				Overall: 1.56 Girls: 1.44 Boys: 1.68	
Rossi ^[64]^ Back pain IR in 1 000 h of AE	Non traumatic: 0.3							
Herbst ^[65]^ Patellofemoral pain IR in 1 000 AE	0.97							0.97
Myer ^[66]^ Patellofemoral pain IR in 100 athletes or in 1 000 AE	9.66 per 100 athletes 1.09 per 1,000 AE							9.66 per 100 athletes 1.09 per 1 000 AE
Foss ^[67]^ Anterior knee pain in %	N= 183 (26.6%)							
Plisky ^[69]^ Lower limb injuries IR in %	23.0%					23.0%		
Wang ^[70]^ Ankle injuries IR in N	N= 18 (42.9%)						N= 18 (42.9%)	
Backman ^[71]^ Patellar tendinopathy IR in N	N= 12 (16.0%)							N= 12 (16.0%)

h of exp., hours of exposure; IR, incidence rate; Ahs, athlete hours; AE, athlete exposure; RR, rate ratio; N, number of participants; exp.,exposure; h, hours; CI, confidence interval

**Table 3:** Musculoskeletal injuries among youth basketball players: preventive intervention and related effectiveness

Reference	Participation and design	Injury definition	Preventive intervention	Outcome
**Foss** ^[72]^	N: 247 G: Girls A: Middle-school and high-school aged. C: US D: RCT F: 1 basketball season.	Injury was defined as: 1. Any injury causing cessation of participation in the current session. 2. Any injury that caused cessation of participation on the day after the day of onset. 3 Any fracture 4 Any dental injury 5 Any mild brain injury.	CORE intervention: The CORE intervention consisted of exercises focused on the trunk and lower extremity, SHAM intervention: The SHAM protocol consisted of resisted running using elastic bands.	The CORE group (rate = 4.99 injuries/1 000 AEs) demonstrated lower injury incidences than the athletes in the SHAM group (rate =7.72 injuries/1 000 AEs) P = 0.002. The absolute risk reduction rate per 1 000 AEs was: 2.73 (95% CI 0.92, 4.54). The CORE group had a reduction in injuries (X^2^ =5.51, P=0.02). A total of 39 of 126 (31%) CORE group athletes and 55 of 121 (45%) SHAM group athletes sustained at least one injury. High school level, 14 of 53 (26%) CORE group athletes versus 17 of 30 (57%) SHAM group athletes incurring an injury (X^2^ = 7.49, P= 0.006). At the middle-school level, the number of injured athletes in the CORE group (25 of 73 [34%] athletes) and the SHAM group (38 of 91 [42%] athletes) was not different (X^2^ = 0.97, P = 0.33).
**Labella** ^[73]^	N: 1492 (soccer and basketball). (755 control group and 737 in intervention group). G: Girls A: High-school age. C: US D: Cluster Randomised Controlled Trial F: Unknown	Lower extremity injuries.	Intervention group: 20-minute neuromuscular warm-up. Control: Control coaches used their usual warm-up.	Intervention athletes had lower rates per 1 000 AEs of gradual-onset LE injuries (0.43 vs 1.22, *P<0*.01), acute onset non-contact LE injuries (0.71 vs 1.61, *P<0*.01), non-contact ankle sprains (0.25 vs 0.74, *P*=0.01), and LE injuries treated surgically (0 vs 0.17, *P*=0.04).
**Emery** ^[74]^	N: 920 Boys n=464 Girls n=456 A: 12–18 years C: Canada D: Cluster Randomised Controlled Trial. F: 1 year		Both groups were taught a standardised warm-up programme. Intervention group: In addition, teams in the training group received an additional five-minute sport-specific balance training warm-up component for practice sessions and a 20-minute home exercise programme using a wobble board.	The programme was protective of acute onset injuries in high school basketball [RR = 0.71 (95% CI; 0.5–0.99)]. The protective effect found with respect to all injury [RR = 0.8 (95% CI; 0.57–1.11)], lower extremity injury [RR = 0.83 (95% CI; 0.57–1.19)], and ankle sprain injury [RR = 0.71 (95% CI; 0.45–1.13)] were not statistically significant. Self-reported compliance to the intended home-based training programme was poor (298/494 or 60.3%). The programme was effective in reducing acute onset injuries in high school basketball. There was also a clinically relevant trend found with respect to the reduction of all lower extremities and ankle sprain injuries.
**McGuine** ^[75]^	N: 1460 (740 braced group and 720 control group. G: Boys and girls A: High-school aged. C: Unknown D: RCT F: One basketball season (2009–2010)	Injury: An event that occurred during a basketball exposure that forced the athlete to stop participation and prevented the athlete from participating in basketball activities the following day.	Intervention group: McDavid Ultralight 195 braces were used. Control: In principle, the control group did not wear an ankle brace.	Acute ankle injury was 68% less in braced group than in control. Acute ankle injury rate braced 0.47/1 000 exposures and control 1.41/1 000 exposures ([HR] 0.32; 95% [CI] 0.20, 0.52; P = <0 .001). For players with a previous ankle injury, the incidence of acute ankle injury was 0.82/1 000 exposures in the braced group and 1.79/1 000 exposures in the control group ([HR] 0.30; 95% CI 0.17,0.90; P = 0.028). For players who did not report a previous ankle injury, the incidence of acute ankle injury was 0.40 in the braced group and 1.35 in the control group ([HR] 0.30; 95% CI 0.17, 0.52, *P* <0.001). The use of a lace-up ankle brace reduced the incidence but not severity of acute ankle injuries in male and female high school basketball athletes by 68% regardless of sex, age, level of competition, or BMI compared with wearing no brace.

N, number of participants; G, gender; A, age; C, country where study was conducted; D, design; F, follow-up period; RCT, randomised controlled trial; AE, athlete exposures; HR, cox hazard ratio; CI, confidence interval; RR, relative risk
