# Supplementary material for: The prevention of injuries among youth basketballers according to the “Sequence of Prevention”: a systematic review
Source: S Afr J Sports Med. 2021 Jan 15;33(1):v33i1a10829. doi: 10.17159/2078-516X/2021/v33i1a10829 (PMC11534288; doi:10.17159/2078-516X/2021/v33i1a10829)
Supplement: Supplementary file 1 [file 2078-516x-33-v33i1a10829-s001.pdf]

The prevention of injuries among youth basketballers according to the "Sequence of Prevention": a systematic review

Appendix 1: Search strategies

Medline via PubMed

#1 = Wounds and Injuries[Mesh] OR injur*[tiab]
#2 = Youth basketball[tiab] OR child[Mesh] basketball[tiab] OR children basketball[tiab] OR adolescent[Mesh] basketball[tiab].
#3 = Cohort studies[mesh:noexp] OR longitudinal studies[mesh:noexp] OR follow-up studies[mesh:noexp] OR prospective studies[mesh:noexp] OR systematic review OR cohort[TIAB] OR longitudinal[TIAB] OR prospective[TIAB] NOT retrospective[TIAB] NOT retrospective studies[mesh:noexp]
#4 (first and second research questions) = #1 AND #2 AND #3 (Filters: Humans; English)
#5 (third and fourth research questions) = #1 AND #2 (Filters: Randomised Controlled Trial; Humans; English)

SPORTDiscus via EBSCOhost

#1 = AB (injur* OR caus* OR epidemiol* OR etiolog* OR etiology* OR mechanism* OR preval* OR inciden* OR occur* OR propor* OR distribut* OR populat* OR risk factor* OR predispose* OR prevent* OR intervent*)
#2 = Youth basketball OR child basketball OR children basketball OR adolescent basketball.
#3 (all four research questions) = #1 AND #2 (Filters:Academic Journal; English)

Appendix 2: Risk of bias appraisal

Quality in Prognosis Studies (QUIPS)

1. Study participation
   - Description of the source population or population of interest
   - Description of the baseline study sample
   - Adequate description of the study sample recruitment (place, period, sampling strategy)
2. Study attribution
   - Adequate response rate for study participants
   - Description of attempts to collect information on participants who dropped out
   - Reasons for loss to follow-up are provided
3. Prognostic factor (PF) measurement
   - A clear definition or description of the PF is provided
   - Method of PF measurement is adequately reliable and valid
4. Outcome measurement
   - A clear definition of the outcome is provided
   - Method of outcome measurement used is adequately reliable and valid
5. Study confounding
   - Clear definition/description of the important confounders measured are provided
   - Measurement of all important confounders is adequately reliable and valid
   - Important potential confounders are accounted for in the analysis
6. Analysis and reporting
   - Sufficient presentation of data to assess the adequacy of the analytic strategy
   - The statistical analyses are adequately reported (without selective reporting)

Cochrane Collaboration's tool

1. Sequence generation
   - Describe the method used to generate the allocation sequence in sufficient detail to allow an assessment of whether it should produce comparable groups.
2. Allocation concealment
   - Describe the method used to conceal the allocation sequence in sufficient detail to determine whether intervention allocations could have been foreseen in advance of, or during, enrolment.
3. Blinding of participants, personnel and outcome assessors
   - Describe all measures used, if any, to blind study participants and personnel from knowledge of which intervention a participant received. Provide any information relating to whether the intended blinding was effective.
4. Incomplete outcome data

- Describe the completeness of outcome data for each main outcome, including attrition and exclusions from the analysis. State whether attrition and exclusions were reported, the numbers in each intervention group (compared with total randomised participants), reasons for attrition/exclusions where reported, and any reinclusions in analyses performed by the review authors.

5. Selective outcome reporting
   - State how the possibility of selective outcome reporting was examined by the review authors, and what was found.
6. Other sources of bias
   - State any important concerns about bias not addressed in the other domains in the tool. If particular questions/entries were prespecified in the review's protocol, responses should be provided for each question/entries.

Appendix 3: Musculoskeletal injuries among youth basketball players: occurence and aetiology

Reference	Participation and design	Injury definition	Incidence	Risk factors
**Leppänen [49]**	N: 201<br>Boys n=100<br>Girls n=101<br>A: 12-20 years<br>C: Finland<br>D: Prospective cohort study<br>F: 3 years	Overuse injury:<br>An injury caused by a repetitive microtrauma and had no single identifiable event causing the injury.	204 overuse injuries in basketball and floorball: (the knee 71 and the lower back 42 overuse injuries)<br>Incidence rate (IR) in 1 000 h of exposure (95% CI)<br>Overall: IR 1.51 (95% CI 1.35 - 1.78)<br>Basketball: IR 1.51 (95% CI 1.20 - 1.82)<br>Basketball girls: IR 1.93 (95% CI 1.43 - 2.56)<br>Basketball boys: IR 1.20 (95% CI 0.86 - 1.62)<br><u>Injury location:</u><br>- Knee 35 0.59 (0.42 to 0.81)<br>- Lower back 20 0.36 (0.21 to 0.51)<br>- Foot 7 0.18 (0.05 to 0.23<br>- Shin/Calf 7 0.18 (0.05 to 0.23)<br>- Hip/Groin 5 0.08 (0.03 to 0.19)<br>- Achilles 6 0.10 (0.04 to 0.21)<br>- Ankle 4 0.07 (0.02 to 0.16)<br>- Thigh 4 0.07 (0.02 to 0.16)<br>- Elbow 1 0.02 (0.00 to 0.08)<br>- Pelvis/Sacrum 1 0.02 (0.00 to 0.08)<br><u>Injury type:</u><br>-Muscle/tendon 54 0.91 (0.69 to 1.17)<br>-Undefined^^ 15 0.25 (0.15 to 0.41)<br>-Joint/ligament 9 0.15 (0.07 to 0.28)<br>-Bone injury 11 0.18 (0.10 to 0.32)<br>-Other 1 0.02 (0.00 to 0.08)	The highest incidences of overuse injuries were registered in April and May during the transition of competition and training seasons.
**Pasanen [50]**	N: 201<br>Boys n=100<br>Girls n=101<br>A: 12-18 years<br>C: Finland<br>D: Prospective study<br>F: 3 years	Injury situations were categorised as "contact," "indirect contact," or "non-contact" injuries.<br>Contact injury:<br>an injury sustained as a result of direct contact with another player or object to the injured body region.<br>Noncontact and indirect contact injury:<br>results from the athlete's own movements without direct contact with injured body part.	158 acute time-loss injuries<br>Incidence rate (IR) in 1 000 h of exposure (95% CI)<br>Overall: IR 2.64 (95% CI 2.23 – 3.05)<br>Ankle injuries games: IR 15.05 (95% CI 9.79 - 20.31)<br>Knee injuries games: IR 6.80 (95% CI 3.25 - 10.34)<br>Girls game-related: IR 32.43 (95% CI 22.01 - 42.85)<br>Boys game-related: IR 36.84 (95% CI 24.86 - 48.82)<br>Girls training-related: IR 1.56 (95% CI 1.06 - 2.05)<br>Boys training-related: IR 1.47 (95% CI 1.06 - 1.88)<br>All (n=201) / Girls (n=101) / Boys (n=100)<br><u>Location:</u><br>Ankle 75 36 39<br>Knee 23 14 9<br>Thigh 11 3 8<br>Finger 10 5 5<br>Lower back 10 4 6<br>Hip/groin 7 2 5<br>Lower leg 5 2 3<br>Upper Back 4 1 3<br>Wrist 3 2 1<br>Foot 2 2 0<br>Shoulder 2 1 1<br>Elbow 1 0 1	Injury situations:<br>- Collision/body contact with other player 25% - Stepping/standing on other player's foot 23%<br>- Landing from a jump 16%<br>- Contact injuries 49%<br>- Indirect contact injuries 17%<br>- Non-contact injuries 34%.<br>- 47% of ankle ligament injuries (n=35) and 95% (n=18) of knee ligament injuries were noncontact/indirect contact injuries.

Appendix 3 continued.

Reference	Participation and design	Injury definition	Incidence	Risk factors
			Injury type:<br>Joint/ligament 106 51 55<br>Muscle/tendon 16 5 11<br>Contusion 13 7 6<br>Undefined 11 4 7<br>Fracture 7 5 2<br><br>Specific injuries:<br>ACL rupture 4 3 1<br>Lateral ankle sprain 72 33 39	
**Kuzuhara [51]**	N: 95<br>Boys n=56<br>Girls n=39<br>A:9-12 years<br>C: Japan<br>D: Descriptive epidemiology study<br>F: 1 season	An injury was defined as any event, including trauma, overuse, or internal disease, related to sports, that conformed to three criteria:<br>1. Occurred during a regular practice or game.<br>2. Led to a player missing any practice or game subsequent to the injury.<br>3 Caused the player to seek medical care from a physician or alternative medical specialist.	Incidence rate (IR) in 1 000 athlete hours (AHs) (95% CI)<br><br>Overall: IR 3.83 (95% CI 3.04 - 3.87)<br>Games: IR 12.92 (95% CI 7.52 - 18.32)<br>Practice: IR 3.13 (95% CI 2.39 - 4.62)<br><br>Injury location:<br>- Head and neck: IR 0.80 (95% CI 0.44 - 1.16)<br>- Upper limb: IR 1.64 (95% CI 1.12 - 2.16)<br>- Trunk and back: IR 0.04 (95% CI 0.00 - 0.12)<br>- Lower limb: IR 0.93 (95% CI 0.54 - 1.32)<br><br>Injury type:<br>- Sprains: IR 1.64 (95% CI 1.12 - 2.16)<br>- Strain: IR 0.08 (95% CI 0.00 - 0.20)<br>- Contusion: IR 1.14 (95% CI 0.71 - 1.57)<br>- Fracture: IR 0.17 (95% CI 0.01 - 0.33)<br>- Osgood-Schlatter: IR 0.13 (95% CI 0.00 - 0.27)	- The resulting considerable differences in height may increase the risk of head and face injuries in short players<br><br>- Most game injuries resulted from body contact (45.5%, 5.87/1 000 AHs), whereas most practice injuries resulted from other contact (56.5%, 1.77/1 000 AHs)
**Rechel [52]**	N: -<br>G: Boys and girls<br>A: High-school age<br>C: US<br>D: Prospective injury surveillance study.<br>F: 1 school year (2005-2006)	An injury was defined as a condition meeting the following 3 criteria:<br>1: Occurred as a result of participation in an organised high school practice or competition.<br>2: Required medical attention by an AT or physician.<br>3: Resulted in restriction of the student-athlete's participation for one day or more beyond the day of injury.	Incidence rate (IR) in 1 000 athlete exposure (AE) (95% CI)<br><br>Girls in competition: IR 3.60 RR 2.63 (95%CI 2.15 - 3.22)<br>Boys in competition: IR: 2.98 RR 2.05 (95%CI 1.69 - 2.49)<br>Girls in practice: IR 1.37<br>Boys in practice: IR 1.46	-Competition.
**Messina [53]**	N: 1863<br>Boys n=973<br>Girls n=890<br>A: 14-18 years<br>C: US<br>D: Prospective study<br>F: a single basketball season	Sprain, contusion, fracture, dislocation, concussion, laceration and dental.	Boys: IR 0.56 per athlete per season.<br>Girls: IR 0.49 per athlete per season.<br>Boys knee injury: IR 0.06 injuries per athlete year.<br>Girls knee injury: 0.1 injuries per athlete year.<br><br>Boys (injury risk) Girls (injury risk)<br>Player hours:<br>Total 169885(3.2) 120751(3.6)<br>Practice 153830(1.8) 107353(2.0)<br>Game 16055(16.9) 13398(16.0)<br><br>Boys(n=543) Incidence(%) Girls(n=436) Incidence(%)<br>Injury type:<br>Sprain 257(47) 243(56)<br>Contusion 108(20) 65(15)<br>Fracture 26(5) 26(6)<br>Dislocation 15(3) 9(2)<br>Laceration 49(9) 8(2)<br>Other 72(13) 71(16)	-In both groups, the most common injuries were sprains, and the most commonly injured area was the ankle, followed by the knee.<br><br>-Female athletes had a significantly higher rate of knee injuries, including a 3.79 times greater risk of anterior cruciate ligament injuries.<br><br>-For both sexes, the risk of injury during a game was significantly higher than during practice.<br><br>-Boys were 1.14 times more likely to sustain an injury than girls.<br><br>-For boys the overall risk of a player sustaining an injury was 3.2 injuries per 1 000 player hours.<br><br>-For girls the overall risk of a player sustaining an injury was 3.6 injuries per 1 000 player hours.

Appendix 3 continued.

Reference	Participation and design	Injury definition	Incidence	Risk factors
			Location:<br>Ankle 137(32) 135(31)<br>Knee 53(10) 86(20)<br>Hip/thigh 55(10) 40(9)<br>Hand/finger 48(9) 35(8)<br>Back 31(6) 27(6)<br>Foot 21(4) 23(5)<br>Shoulder 24(4) 12(3)<br>Leg 24(4) 19(4)<br>Arm/wrist 19(3) 10(2)<br><br>ACL injury:<br>Incidence 4(0.007) 11(0.025)<br>Rate 0.004 0.012<br>Risk 0.024 0.09	
**Gomez [54]**	N: 890<br>G: Girls<br>A: 14-18 years<br>C: US<br>D: Prospective cohort<br>F: 1 season (1993/1994)	Occurred in a practice or a game, from the start of pre-season training to the completion of post-season play, in which the injury resulted in either missed practice or game time, necessitated the consultation of a physician, or involved the head or face.	-The overall injury rate was 0.49 per athlete per season.<br>-The risk of a player sustaining an injury was 0.004 or 0.4% per hour of exposure.<br>-The rate of serious injury was 0.038 per athlete per season.<br>-The risk calculated using team exposure hours is 0.0035 serious injuries per team per hour.<br><br>Injury No.(%)<br>Sprain/strain 243 (56)<br>Constusion 65 (15)<br>Fracture 26 (6)<br>Dislocation 9 (2)<br>Laceration 8 (2)<br><br>Location No.(%)<br>Ankle 135 (31)<br>Knee 86 (19)<br>Hip/thigh 40 (9)<br>Hand/fingers 35 (8)<br>Back 27 (6)<br>Foot 23 (5)<br>Leg 19 (4)<br>Shoulder 12 (3)<br>Arm/wrist 10 (2)<br>Chest 4 (1)	
**Backx [56]**	N: 36<br>G: Boys and girls<br>A: 8 -17 years<br>C: Netherlands<br>D: Longitudinal study<br>F: 7 months	Contusion, sprain, strain, fracture/dislocation, abrasion/laceration, chondromalacia patellae, inflammation, concussion and uncertain diagnosis.	-Incidence rate in organized sports and physical education: 998 per 1 000 young athletes a year.<br>-Incidence rate in games: 23 injuries per 1 000 hours.	-Game situations.<br><br>-Incidence = -919.4 +35.3 outdoor + 51.6 high jump +55.3 contact, explaining 78% of the total variance.
**Yde [56]**	N: 56<br>Boys n=27<br>Girls n=29.<br>A: <10 y (9), <14 y(13) and <18y (34).<br>C: Denmark<br>D: Prospective study<br>F: One season.	An injury was defined as an incident occurring during a match or training in the club, causing the player to miss at least one match or one training session.	Incidence rate (IR) per 1 000 playing hours<br><br>Basketball injuries: IR 3.0<br>Practice: IR 2.4<br>Games: IR 5.7<br><br>Percentage of different injuries:<br>Hand/fingers: 43%<br>Ankle: 33%<br>Knee: 5%<br>Thigh/leg: 5%<br>Foot 0%<br>Shoulder/arm: 0%<br>Other: 14%	-Ball contact, running and shooting are the most significant injury situations.
**Owoeye [57]**	N: 141<br>Boys n=75<br>Girls n= 66<br>A: 15-18 years.<br>C: Nigeria<br>D: A prospective observational study<br>F: 32 matches	An injury was documented when an injured player required at least minimum on-field (medical) care such as ice, tape, etc. regardless of whether the player was able to continue or not.	Overall for both genders: IR 22.7 per 100 participants.<br><br>Incidence rate (IR) per match<br><br>Overall: IR 1.0 per match.<br>Boys: IR 1.1 per match.<br>Girls: IR 0.9 per match.<br><br>-Sprain was found to be the most common type of injury accounting 62.5% of all injuries.	- Jumping/landing was found to be the most common cause of injury (28.1%).

Appendix 3 continued.

Reference	Participation and design	Injury definition	Incidence	Risk factors
			-The knee was the most commonly affected body part, accounting for 13 cases (40.6%), followed by the ankle (N=7, 21.9%).<br><br>Boys n(%) / Girls n(%) / Total n(%)<br>Contusion 2(66.7) 1(33.3) 3(9.4)<br>Dislocation 1(100.0) 0(0.0) 1(3.1)<br>Laceration 1(50.0) 1(50.0) 2(6.3)<br>Sprain 11(55.0) 9(45.0) 20(62.5)<br>Strain 2(40.0) 3(60.0) 5(15.6)<br>Cramp 0(0.0) 1(100.0) 1(3.1)<br><br>Face 2(100.0) 0(0.0) 2(6.3)<br>Abdomen 0(0.0) 1(100.0) 1(3.1)<br>Forearm 1(100.0) 0(0.0) 1(3.1)<br>Wrist&finger 1(100.0) 0(0.0) 1(3.1)<br>Elbow 1(33.3) 2(66.7) 3(9.4)<br>Hip&Thigh 0(0.0) 1(100.0) 1(3.1)<br>Leg 0(0.0) 1(100.0) 1(3.1)<br>Knee 7(53.8) 6(46.2) 13(40.6)<br>Ankle 4(57.1) 3(42.9) 7(21.9)<br>Toes 1(50.0) 1(50.0) 2(6.3)	
**Clifton [58]**	N: -<br>G: Boys<br>A: High-school aged<br>C: US<br>D: Descriptive epidemiology study<br>F: In the 2005-2006 through 2013-2014 academic years.	A reportable injury was defined as an injury that:<br>1: Occurred as a result of participation in an organised practice or competition.<br>2: Required medical attention by a certified AT or physician.<br>3: Resulted in restriction of the student-athlete's participation for one or more days beyond the day of injury.	- The total injury rate for high school boys' basketball was 1.55/1 000 AEs.<br>- Injury rates, overall as well as by body part and specific diagnosis, were greater during competitions than practices (overall IRR = 2.38; 95% CI = 2.22, 2.56)<br><br><u>Body Part Injured</u> / Injury Rate Practice 1 000 AEs / Injury Rate Competition 1 000 AEs<br>Neck <0.01 0.01<br>Shoulder/clavicle 0.03 0.09<br>Arm/elbow 0.02 0.08<br>Hand/wrist 0.11 0.21<br>Trunk 0.06 0.12<br>Hip/thigh/upper leg 0.07 0.17<br>Knee 0.12 0.33<br>Lower leg 0.04 0.06<br>Ankle 0.39 0.85<br>Foot 0.06 0.11<br><br><u>Diagnosis</u> / Injury Rate Practice 1 000 AEs / Injury Rate Competition 1 000 AEs<br>Contusion 0.07 0.3<br>Dislocation 0.03 0.06<br>Fracture/avulsion 0.12 0.32<br>Laceration 0.04 0.1<br>Ligament sprain 0.47 1.07<br>Muscle/tendon strain 0.15 0.22<br><br><u>Mechanism</u> / Injury Rate Practice 1 000 AEs / Injury Rate Competition 1 000 AEs<br>Contact with another 0.47 1.32<br>Contact with playing surface 0.20 0.68<br>Contact with ball 0.06 0.05<br>Contact with other playing equipment 0.01 0.02<br>Contact with out-of-bounds object <0.01 <0.01<br>No contact 0.23 0.43<br>Overuse/chronic 0.08 0.04<br>Illness/infection 0.02 <0.01	Division I had a higher total injury rate than Division II (IRR = 1.17; 95% CI = 1.08, 1.27) but not Division III (IRR = 1.04; 95% CI = 0.97, 1.11). Also, Division III had a higher total injury rate than Division II (IRR = 1.12; 95% CI = 1.03, 1.22).

Appendix 3 continued.

Reference	Participation and design	Injury definition	Incidence	Risk factors
			<u>Activity</u> — Injury Rate Practice 1 000 AEs / Injury Rate Competition 1 000 AEs<br>Ball handling 0.05 0.18<br>Conditioning 0.08 0.33<br>Defending 0.07 0.01<br>General play 0.15 0.46<br>Loose ball 0.24 0.34<br>Passing 0.01 0.01<br>Rebounding 0.27 0.71<br>Receiving pass 0.05 0.07<br>Screening 0.01 0.02<br>Shooting 0.09 0.34	
**Clifton [59]**	N: -<br>G: Girls<br>A: High-school aged<br>C: US<br>D: Descriptive epidemiology study<br>F: In the 2005-2006 through 2013-2014 academic years.	A reportable injury was defined as an injury that (1) occurred as a result of participation in an organized practice or competition; (2) required medical attention by a certified AT, physician, or other health care professional; and (3) resulted in restriction of the student-athlete's participation for one or more days beyond the day of injury.	- The total injury rate for high school girls' basketball was 1.82/1 000 AEs.<br>- Injury rates, overall as well as by body part and specific diagnosis, were greater during competitions than practices (overall IRR = 3.03; 95% CI = 2.82, 3.26)<br><br><u>Body Part Injured</u> — Injury Rate Practice 1 000 AEs / Injury Rate Competition 1 000 AEs<br>Neck 0.01 0.01<br>Shoulder/clavicle 0.04 0.12<br>Arm/elbow 0.01 0.06<br>Hand/wrist 0.11 0.26<br>Trunk 0.06 0.11<br>Hip/thigh/upper leg 0.11 0.11<br>Knee 0.19 0.66<br>Lower leg 0.08 0.08<br>Ankle 0.33 0.98<br>Foot 0.06 0.07<br><br><u>Diagnosis</u> — Injury Rate Practice 1 000 AEs / Injury Rate Competition 1 000 AEs<br>Contusion 0.06 0.26<br>Dislocation 0.02 0.07<br>Fracture/avulsion 0.07 0.25<br>Laceration <0.01 0.05<br>Ligament sprain 0.45 1.44<br>Muscle/tendon strain 0.18 0.34<br><br><u>Mechanism</u> — Injury Rate Practice 1 000 AEs / Injury Rate Competition 1 000 AEs<br>Contact with another 0.31 1.55<br>Contact with playing surface 0.19 0.87<br>Contact with ball 0.09 0.13<br>Contact with other playing equipment 0.01 0.01<br>Contact with out-of-bounds object <0.01 0.01<br>No contact 0.31 0.64<br>Overuse/chronic 0.15 0.06<br>Illness/infection 0.02 0.02<br><br><u>Activity</u> — Injury Rate Practice 1 000 AEs / Injury Rate Competition 1 000 AEs<br>Ball handling 0.06 0.29<br>Conditioning 0.13 0.01<br>Defending 0.12 0.64<br>General play 0.31 0.61	-Playing competition.

Appendix 3 continued.

Reference	Participation and design	Injury definition	Incidence	Risk factors
			Activity / Injury Rate Practice 1 000 AEs / Injury Rate Competition 1 000 AEs<br><br>Loose ball 0.08 0.49<br>Passing 0.02 0.06<br>Rebounding 0.18 0.72<br>Receiving pass 0.08 0.14<br>Screening <0.01 0.01<br>Shooting 0.06 0.24	
**Robinson [60]**	N: ?<br>G: Boys and girls<br>A: High-school aged.<br>C: US<br>D:<br>F: 6 academic years	A shoulder injury was defined as any injury to the proximal humerus, scapula, clavicle, acromioclavicular joint, and surrounding tendons, ligaments, and musculature.	Incidence rate (IR) per 10 000 athlete exposure (AEs)<br><br>Boys overall: IR 0.50<br>Boys competition: IR 0.95<br>Boys practice: IR 0.32<br>Girls overall: IR 0.61<br>Girls competition: IR 1.24<br>Girls practice: IR 0.34	
**Bonza [61]**	N: -<br>G: Boys and girls.<br>A: High-school age<br>C: US<br>D: Prospective injury surveillance study<br>F: During the 2005–2006 and 2006–2007 school years	A shoulder injury met all of the following criteria:<br>1: Occurred as a result of participation in an organised high school practice or competition.<br>2: Required medical attention by an AT or a physician.<br>3 Resulted in restriction of the student-athlete's participation for at least one day beyond the day of the injury.	Incidence rate (IR) per 10 000 athlete exposure (AEs)<br><br>Girls overall: IR 0.45<br>Boys overall: IR 0.47<br>Girls competition: IR 0.76<br>Boys competition: IR 0.90<br>Girls practice: IR 0.32<br>Boys practice: IR 0.30.	-Consistent with prior research, shoulder injury rates in soccer, basketball, and baseball/softball were higher among boys than girls (0.95 and 0.61 per 10,000 AEs, respectively).<br><br>-Competition
**Beynnon [62]**	N: 901 (soccer, basketball, lacrosse and field hockey).<br>Basketball n=159<br>Boys n=68<br>Girls n=91<br>A: High-school age college age.<br>C: US<br>D: Cohort study<br>F:4 years, between 1999 and 2003.	An ankle injury was defined as inversion trauma that forced the athlete to miss at least one practice or game.	Incidence rate (IR) per 1 000 person-days<br><br>Boys: IR 0.42<br>Girls: IR 1.90	-The incidence rate of inversion injury is less than 1 per 1 000 days of exposure to sport<br><br>-Risk is highest for female basketball athletes, who are at significantly greater risk than male basketball athletes.
**McGuine [63]**	N: 210<br>Boys n=119<br>Girls n=91<br>A: 15-18<br>C: US<br>D: Cohort study<br>F: 2 basketball seasons	Ankle sprain injury: Trauma that disrupts the structures of the ankle that occurs during a team-sponsored practice or competition session, and causes the athlete to miss the rest of practice or competition or miss the next scheduled team practice or competition.	Incidence rate (IR) per 1 000 exposure<br><br>Overall: IR 1.56<br>Boys: IR 1.68<br>Girls: IR 1.44	-Higher postural sway scores corresponded to increased ankle sprain injury rates (p = 0.001).<br><br>-Subjects who demonstrated poor balance (high sway scores) had nearly seven times as many ankle sprains as subjects who had good balance (low sway scores) (p = 0.0002.)
**Rossi [64]**	N: 396 (basketball and floorball players).<br>G: Boys and girls<br>A: 15.8 +/ 1.9 years<br>C: Finland<br>D: Prospective follow-up study.<br>F: 3 years	Back pain:<br>Pain in the upper and/or lower back area, that prevented the player from fully participating in the team training and playing during the following 24 hours.	Incidence rate (IR) per 1 000 h of AE<br><br>Floorball and basketball: IR 0.4<br>Non-traumatic back pain in basketball: IR 0.3<br><br>Total number (%)<br><br>Low back/pelvis<br>Non-traumatic 17(70.8)<br>Acute traumatic 6 (25.0)<br>Total 23(95.8)<br><br>Upper back<br>Non-traumatic 0(0.0)	-Lower extremity muscle extensibility, general joint hypermobility or investigated lower extremity strength measures were not associated with the risk of LBP.

Appendix 3 continued.

Reference	Participation and design	Injury definition	Incidence	Risk factors
			Acute traumatic 1(4.2)<br>Total 1(4.2)<br><br>All<br>Non-traumatic 17(70.8)<br>Acute traumatic 7 (29.2)<br>Total 24(100.0)	
**Herbst [65]**	N: 329<br>G: Girls<br>A: Adolescent age<br>C: US<br>D: Cohort<br>F: 1 basketball season	Patellofemoral pain (PFP).	-The cumulative incidence rate for the development of PFP, unilateral or bilateral, was 0.97 per 1 000 AEs.	-Young female athletes with greater hip abduction strength may be at an increased risk for the development of PFP.
**Myer [66]**	N: 240<br>G: Girls<br>A: Middle- and high-school aged. (mean age: 13.4 years).<br>C: US<br>D: Cohort<br>F: 1 competitive season.	Subjects were diagnosed as having active PFP if they presented with AKPS score < 100.	-At the beginning of the season, the point prevalence of PFP was 16.3 per 100 athletes.<br>-The cumulative incidence risk and rate for the development of new unilateral PFP was 9.66 per 100 athletes and 1.09 per 1 000 athletic exposures.	-The increased knee abduction landing mechanics in the new PFP group indicate that frontal plane loads contribute to increased incidence of PFP.
**Foss [67]**	N: 419<br>G: Girls<br>A: Middle and high school-aged.<br>C: US<br>D: Descriptive epidemiology study.<br>F: 3 basketball seasons.	Anterior knee pain.	688 examinations, 183 (26.6%) were positive for anterior knee pain. Prevalence between school levels differed, with 34.4% (67 cases) in high-school aged athletes (n = 195) versus 23.5% (116 cases) in middle-school aged athletes (n = 493, P < .05)<br><br>Diagnosis High School (%) Middle School (%)<br>Patellofemoral dysfunction 26(6.7) 74(7.5)<br>Sinding-Larsen-Johansson 38(9.7) 31(3.1)<br>Osgood-Schlatter 7(1.8) 24(2.4)<br>Plica 10(2.6) 19(1.9)<br>Trauma 4(1) 11(1.1)<br>Fat pad irritation of inflammation 2(0.5) 3(0.3)<br>Illiotibial band tightness 1(0.3) 3(0.3)<br>Pes anserine bursitis 1(0.3) 0(0)	-Symptoms of anterior knee pain likely persist after middle school-aged onset and reach peak prevalence during the high school years.
**Field [68]**	N: 6831<br>G: Girls<br>A: 9-15 years at baseline.<br>C: US<br>D: Prospective cohort study<br>F: 7 years	When a doctor has ever said that their child had a stress fracture and when the mother indicated that her child had a history of stress fracture.	Hazard ratio, adjusted for age and family history (95% CI) / HR, adjusted for all covariates in model (95% CI)<br>Basketball (hrs/wk) 1.19 (1.10-1.28) 1.12 (1.03-1.23)	-Females reporting a family history were almost two times more likely to develop a stress fracture (OR=1.95, 95% CI 1.24–3.07).<br>-Girls who engaged in eight or more hours of activity per week were twice as likely as their peers who engaged in less than four hours of activity to develop a stress fracture.
**Plisky [69]**	N: 235<br>Boys n=130<br>Girls n= 105<br>A: High-school age.<br>C: US<br>D: Prospective Cohort.<br>F:1 basketball season (2004-2005).	Lower limb injuries.	-23.0% incurred a lower extremity injury.<br>-92.5% of the injuries were traumatic in nature and four injuries were considered overuse-related injuries.	-Players with a greater anterior right/left reach distance difference were 2.5 times more likely to sustain a lower extremity injury.<br>-For all players, anterior right/left reach distance difference greater than or equal to four cm, decreased normalised right anterior reach distance, and decreased normalised posteromedial, posterolateral, and composite reach distances bilaterally were significantly associated with lower extremity injury ($P<0.05$).

Appendix 3 continued.

Reference	Participation and design	Injury definition	Incidence	Risk factors
**Wang [70]**	N:42<br>G: Boys<br>A: 16.5 +/ 1.1 years.<br>C: Taiwan<br>D: Cohort study<br>F: 1 basketball season.	Ankle injuries.	n Ankle injured (n) Non-ankle injured (n) 42 18 24	-High variation of postural sway in both anteroposterior and mediolateral directions corresponded to occurrences of ankle injuries (*P*<0.01, [OR]=1.220; *P*<0.001, OR=1.216, respectively). All other variables were not associated with injury.<br>-High variations of postural sway in one-leg standing test could explain partly the increased prevalence of ankle injury in basketball players.
**Backman [71]**	N: 75<br>Boys n = 38<br>Girls n = 37<br>A: 14-20 years (mean 17.8 +/- 1.6)<br>C: Sweden<br>D: Prospective cohort.<br>F: 1 year.	The following criteria were used for diagnosing patellar tendinopathy:<br>-History of activity-related anterior knee pain and reduced function of the knee.<br>-Distinct palpation tenderness corresponding to the painful area.<br>-Knee pain provoked by a previously described single-legged decline squat test, designed to optimise the loading on the patellar tendon.	- 12 players (16.0%) had developed unilateral PT.	-Players with dorsiflexion range less than 36.5 degrees had a risk of 18.5% to 29.4% of developing PT within a year, as compared with 1.8% to 2.1% for players with dorsiflexion range greater than 36.5 degrees.<br>- Players were found to have had a significantly lower mean ankle dorsiflexion range at baseline than the healthy players, with a mean difference of -4.7 degrees (P = 0.038) for the dominant limb and -5.1 degrees (P = 0.024) for the non-dominant limb.

N, number of participants; G, gender; A, age; C, country where study was conducted; D, design; F, follow-up period; h, hours; ?, unknown; IR, incidence rate; RR, rate ratio; AE, athlete exposures; OR, odds ratio; CI, confidence interval

Appendix 4: Musculoskeletal injuries among youth basketball players: preventive interventions and related effectiveness

Reference	Participation and design	Injury definition	Preventive intervention	Outcome
**Foss [72]**	N: 247<br>G: Girls<br>A: Middle-school and high-school aged.<br>C: US<br>D: RCT<br>F: 1 basketball season.	Injury was defined as:<br>1. Any injury causing cessation of participation in the current session.2. Any injury that caused cessation of participation on the day after the day of onset.<br>3. Any fracture<br>4. Any dental injury<br>5. Any mild brain injury.	From the first day of team practice until the first competition, teams were instructed to perform the training for 20 to 25 minutes, three times per week. When competition started, teams were instructed to perform a reduced-volume training protocol for 10 to 15 minutes, two times per week, until the end of the competitive season.<br><br><u>CORE intervention:</u><br>Core exercises pre-season:<br>-Lateral jump and hold eight repetitions on each lower limb<br>-Step hold eight repetitions on each lower limb<br>-BOSUa (round) swimmers two sets of 10 repetitions<br>-BOSUa (round) double-knee hold Two 20-s repetitions<br>-Single-legged lateral AIREXb hop-hold 4 repetitions on each lower limb<br>-Single tuck jump with soft landing two sets of 10 repetitions<br>-Front lunges 10 repetitions on each lower limb<br>-Lunge jumps 10 repetitions on each lower limb<br>-BOSUa (flat) double-legged pelvic bridges two sets of 10 repetitions<br>-Single-legged 908 hop-hold eight repetitions on each lower limb<br>-BOSUa (round) lateral crunch 10 repetitions on each lower limb<br>-Box double crunch two sets of 15 repetitions<br>-Swiss ball back hyperextensions two sets of 15 repetitions.<br><br><u>Core exercises in-season:</u><br>Step hold eight repetitions on each lower limb<br>-BOSUa (flat) double-legged pelvic bridges two sets of eight repetitions<br>-Single-legged 908 hop-hold 10 repetitions on each lower limb<br>-Single-legged Romanian dead lift one set on each lower limb<br>-Unanticipated hop to stabilisation (level 1) Three five repetitions on each lower limb<br>-Hop to stabilisation and reach (level 1) three repetitions on each lower limb<br>-Single tuck jump with soft landing Two 10-s repetitions<br><br><u>SHAM intervention:</u><br>The SHAM protocol consisted of resisted running using elastic bands.	- For basketball, the athletes in the CORE group (rate = 4.99 injuries/1 000 AEs) demonstrated lower injury incidences than the athletes in the SHAM group (rate =7.72 injuries/1 000 AEs) P = 0.002. The absolute risk reduction rate per 1 000 AEs was: 2.73 (95% CI 0.92, 4.54).<br><br>-The CORE group had a reduction in injuries (X^2 =5.51, P=0.02). A total of 39 of 126 (31%) CORE group athletes and 55 of 121 (45%) SHAM group athletes sustained at least one injury.<br><br>-High school level, 14 of 53 (26%) CORE group athletes versus 17 of 30 (57%) SHAM group athletes incurring an injury (X^2 = 7.49, P= 0.006).<br><br>-At the middle school level, the number of injured athletes in the CORE group (25 of 73 [34%] athletes) and the SHAM group (38 of 91 [42%] athletes) was not different (X^2 = 0.97, P = 0.33).
**Labella [73]**	N: 1492 (soccer and basketball). (755 control group and 737 in intervention group).<br>G: Girls<br>A: High-school age.<br>C: US<br>D: Cluster Randomised Controlled Trial<br>F: ?	Lower extremity injuries.	<u>Intervention group:</u><br>20-minute neuromuscular warm-up:<br><br>Similar to previously studied NMT programmes, combining progressive strengthening, plyometric, balance, and agility exercises. Athletes were instructed to avoid dynamic knee valgus and to land jumps with flexed hips and knees. We taught coaches how to distinguish proper from improper form and how to use verbal cues to promote proper form (e.g. 'land softly' and 'don't let knees cave inward') because research shows that this feedback enhances effectiveness. Coaches received a DVD with narrated videos of the exercises, a laminated card listing the order and frequency of exercises for use on the court or field, and printed educational materials about knee injury risk factors and neuromuscular exercises<br><br><u>Control:</u><br>Control coaches used their usual warm-up.	Noncontact LE injury rates<br><br>Control group<br>Injury type:<br>Gradual onset 1.22<br>Acute onset 1.61<br>Ankle sprains 0.74<br>Knee sprains 0.48<br>ACL sprains 0.26<br>LE injuries<br>Treated surgically 0.17<br><br>Intervention group<br>Injury type:<br>Gradual onset 0.43<br>Acute onset 0.71<br>Ankle sprains 0.25<br>Knee sprains 0.21<br>ACL sprains 0.07<br>LE injuries<br>Treated surgically 0

Appendix 4 continued.

Reference	Participation and design	Injury definition	Preventive intervention	Outcome
				- Intervention athletes had lower rates per 1 000 AEs of gradual-onset LE injuries (0.43 vs 1.22, *P*<0.01), acute-onset noncontact LE injuries (0.71 vs 1.61, *P*<0.01), noncontact ankle sprains (0.25 vs 0.74, *P*=0.01), and LE injuries treated surgically (0 vs 0.17, *P*=0.04).<br>-Coach-led neuromuscular warm-up reduces noncontact LE injuries in female high school soccer and basketball athletes from a mixed-ethnicity, predominantly low-income, urban population.
**Emery [74]**	N: 920<br>Boys n=464<br>Girls n=456<br>A: 12-18 years<br>C: Canada<br>D: Cluster Randomised Controlled Trial.<br>F: 1 year		Both groups were taught a standardised warm-up programme. A warm-up routine was taught to all of the coaches and teams by an independent study physiotherapist or Certified Athletic Therapist who was not the team therapist. The 10 minute warm-up routine included aerobic, static stretch, and dynamic stretch components. This was considered the 'current standard of practice' for a high school basketball warm-up routine.<br>Intervention group:<br>In addition, teams in the training group received an additional five-minute sport-specific balance training warm-up component for practice sessions and a 20-minute home exercise programme using a wobble board.	-A basketball-specific balance training programme was protective of acute-onset injuries in high school basketball [RR = 0.71 (95% CI; 0.5–0.99)].<br>-The protective effect found with respect to all injury [RR = 0.8 (95% CI; 0.57–1.11)], lower-extremity injury [RR = 0.83 (95% CI; 0.57–1.19)], and ankle sprain injury [RR = 0.71 (95% CI; 0.45–1.13)] were not statistically significant<br>-Self-reported compliance to the intended home-based training programme was poor (298/494 or 60.3%).<br>-A basketball-specific balance training programme was effective in reducing acute-onset injuries in high school basketball. There was also a clinically relevant trend found with respect to the reduction of all, lower extremity, and ankle sprain injury.
**McGuine [75]**	N: 1460 (740 braced group and 720 control group.<br>G: Boys and girls<br>A: High-school aged.<br>C: -<br>D: RCT<br>F: 1 basketball season (2009-2010)	Injury:<br>An event that occurred during a basketball exposure that forced the athlete to stop participation and prevented the athlete from participating in basketball activities the following day.	Intervention group:<br>McDavid Ultralight 195 braces were used. Athletes were instructed to wear the brace over a single pair of socks on both ankles for each team-organised conditioning session, practice, or competition throughout the season.<br>Control:<br>In principle, the control group did not wear an ankle brace. Ankle brace compliance and the use of other external support by control participants (lace-up brace, hard-shell brace, adhesive tape) were monitored by the on-site AT.	-Acute ankle injury was 68% less in braced group than in control.<br>- Acute ankle injury rate braced 0.47/1,000 exposures and control 1.41/1 000 exposures ([HR] 0.32; 95% [CI] 0.20, 0.52; P = < 0.001).<br>-For players with a previous ankle injury, the incidence of acute ankle injury was 0.82/1 000 exposures in the braced group and 1.79/1 000 exposures in the control group ([HR] 0.30; 95% CI 0.17,0.90; P = 0.028).<br>- For players who did not report a previous ankle injury, the incidence of acute ankle injury was 0.40 in the braced group and 1.35 in the control group ([HR] 0.30; 95% CI 0.17, 0.52, *P* <0.001).<br>-The use of a lace-up ankle brace reduced the incidence but not severity of acute ankle injuries in male and female high school basketball athletes by 68% regardless of sex, age, level of competition, or BMI compared with wearing no brace.

N, number of participants; G, gender; A, age; C, country where study was conducted; D, design; F, follow-up period; ?, unknown; RCT, randomised controlled trial; AE, athlete exposures; HR, cox hazard ratio; CI, confidence interval; RR, relative risk

Appendix 5: The most important outcome measures displayed visually.

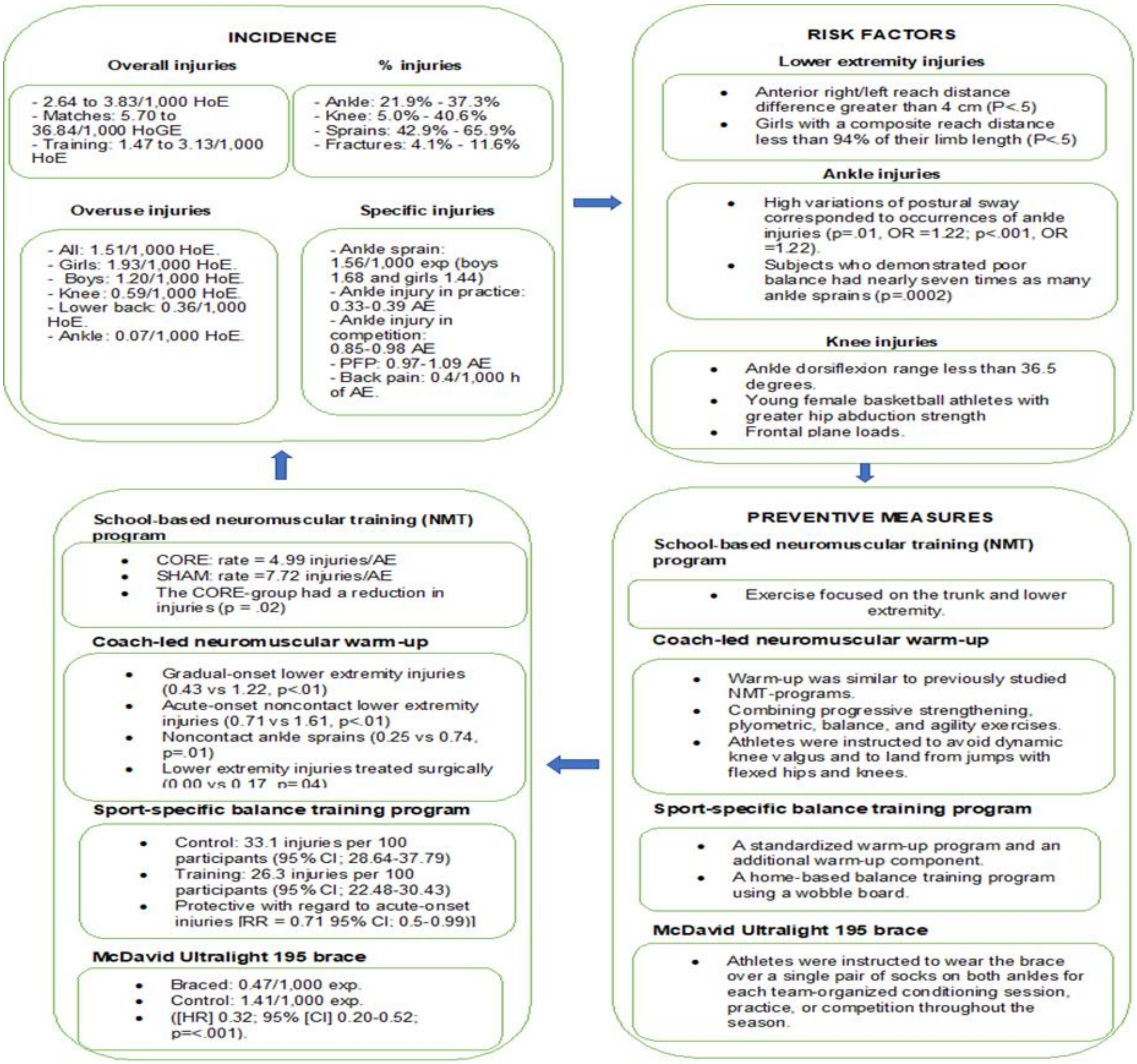

HoE, hours of exposure; HoGE, hours of game exposure; h, hours; exp, exposure; AE, athlete exposure; OR, odds ratio; CI, confidence interval; HR, hazard ratio; RR, relative risk
